# Hepatoprotective Role of 4-Octyl Itaconate in Concanavalin A-Induced Autoimmune Hepatitis

**DOI:** 10.1155/2022/5766434

**Published:** 2022-03-11

**Authors:** Wenchang Yang, Yaxin Wang, Peng Zhang, Tao Wang, Chengguo Li, Xin Tong, Xiangyu Zeng, Yuping Yin, Kaixiong Tao, Ruidong Li

**Affiliations:** ^1^Department of Gastrointestinal Surgery, Union Hospital, Tongji Medical College, Huazhong University of Science and Technology, Wuhan 430022, China; ^2^Department of Critical Care Medicine, Union Hospital, Tongji Medical College, Huazhong University of Science and Technology, Wuhan 430022, China

## Abstract

4-Octyl itaconate (OI) is a novel anti-inflammatory metabolite that exerts protective effects in many various disease models. However, its function in autoimmune hepatitis- (AIH-) associated hepatic injury has not been investigated. In this study, we successfully used concanavalin A (Con A) to establish an AIH-associated liver injury model. Furthermore, we investigated the effect of OI in Con A-induced liver injury and found that OI mitigated Con A-induced histopathological damage. OI administration reduced serum levels of alanine transaminase and aspartate transaminase in Con A-treated mice and attenuated the infiltration of macrophages induced by Con A. Moreover, OI effectively inhibited the expression of proinflammatory cytokines including interleukin-6 (IL-6), tumor necrosis factor-alpha (TNF-*α*), interferon-gamma (IFN-*γ*), and IL-1*β* induced by Con A. Furthermore, OI decreased hepatocyte apoptosis and malondialdehyde levels and increased the reduced glutathione/oxidized glutathione ratio in the Con A-induced liver injury model. In addition, we found that OI inhibited Con A-induced hepatocyte apoptosis in vitro, while Nrf2 deletion eliminated this effect. Furthermore, we administrated the Nrf2 inhibitor ML385 in OI+Con A-treated mice and found that ML385 eliminated the protective effect of OI in vivo. In addition, OI inhibited Con A-induced activation of nuclear factor-kappa B (NF-𝜅B) and the expression of proinflammatory cytokines in macrophages. Therefore, OI protected mice from Con A-induced liver damage and may be associated with Nrf2 activation and NF-𝜅B inhibition. Finally, our study revealed that OI inhibited TNF-*α*, or supernatants from Con A-treated RAW264.7 cells induced hepatocyte apoptosis. In conclusion, our study indicated that OI alleviated Con A-induced hepatic damage by reducing inflammatory response, oxidative stress, and apoptosis.

## 1. Introduction

Acute hepatitis is a common and serious health issue worldwide [1]. Multiple pathogenic factors including toxins, organisms, and autoimmune diseases can lead to hepatic injury and probably incur fulminant hepatitis [2–5]. The pathology of numerous hepatic diseases is involved in inflammatory response, oxidative stress, and hepatocytes death [6–8]. Self-restricted inflammation is normally considered beneficial for fighting pathogens and maintaining hepatic homeostasis [9]. However, excessive inflammation causes massive apoptosis/necroptosis of hepatocytes and leads to destruction of the tissue architecture, which further induces the loss of hepatic function [10]. Auto-immune hepatitis (AIH) is considered immune disorder-induced hepatic injury mediated by abnormal activation of immunocytes and production of proinflammatory mediators [11]. Concanavalin A- (Con A-) induced hepatitis has similar histopathological features and is widely used to establish AIH models in studies [12, 13]. Con A can activate the Kupffer cells (KCs) and promote them to secrete proinflammatory cytokines, such as tumor necrosis factor-alpha (TNF-*α*), thus aggravating the inflammatory response and eventually causing hepatic damage [14, 15]. Moreover, KCs can increase oxidative stress and cause tissue injury during hepatitis [16]. Therefore, targeting macrophages in the liver may be a potential therapeutic approach to treat AIH.

Multiple physiological metabolites in macrophages have exhibited immunoregulation properties and exerted protective effects in various disease models. Itaconate, produced from citrate, is one of the most abundant metabolites in activated macrophages [17]. It exerts protective effects in several inflammation-associated diseases by inhibiting excessive inflammation [18]. Under a certain stimulus, citrate is first catalyzed and transformed to cis-aconitate by aconitate hydratase 2. Subsequently, cis-aconitate is transformed to itaconate by cis-aconitate decarboxylase. Furthermore, 4-octyl itaconate (OI), a cell-permeable derivative of itaconate, exhibits anti-inflammatory activity by activating nuclear factor-erythroid 2-related factor 2 (Nrf2), which is a pivotal transcription factors that regulate multiple antioxidant genes such as heme oxygenase-1 (HO-1) [19]. It can bind with the genes that have the antioxidant response element- (ARE-) like sequences in their regions and promote the elimination of reactive oxygen species (ROS) to exert protective effects [20]. It has been reported that Nrf2 protects normal cells from DNA damage by reducing ROS and protects tumor cells against the chemotherapy [3, 21]. Moreover, Nrf2 was recently found to bind with the promoter regions of some proinflammatory cytokines such as interlukin-6 (IL-6) and directly inhibit their transcription to alleviate the inflammatory response [22]. Previous studies have confirmed that Nrf2 activation played an important role in alleviating liver injury [23, 24]. For example, we previously found that Nrf2 activation mitigated carbon tetrachloride-induced hepatic injury by inhibiting oxidative stress and inflammation [24]. Furthermore, OI mitigated ischemia-reperfusion injury-induced liver injury by activating the Nrf2 pathway [25]. However, the effect of OI on AIH has not yet been investigated.

In this study, we investigated the effect of OI on a Con A-induced AIH model and found that OI protected mice from Con A-induced liver injury. Furthermore, OI administration in Con A-induced AIH reduced hepatocyte death, inflammation, and oxidative stress. The possible underlying mechanism involves the activation of Nrf2 signaling and inhibition of NF-*κ*B signaling. Therefore, our current study found that OI might be a potential therapeutic strategy for AIH.

## 2. Materials and Methods

### 2.1. Reagent

Con A was bought from Sigma Aldrich (St. Louis, MO, USA). 4-OI and ML385 were obtained from Med Chem Express (USA). The alanine transaminase (ALT), aspartate transaminase (AST), and reduced glutathione/oxidized glutathione (GSH/GSSG) assay kit were purchased from Nanjing Jiancheng Institute of Biotechnology (Nanjing, China). Fetal bovine serum and high glucose Dulbecco's modified Eagle's medium (DMEM) were purchased from Gibco Life Technologies (Carlsbad, CA, USA). Murine-TNF-*α* was purchased from PeproTech (USA). The antibodies used in this study include those against HO-1 (Proteintech, Wuhan, China), Nrf2 (Cell Signal Technology, MA, USA), Bax (Proteintech, Wuhan, China), Bcl-2 (Proteintech, Wuhan, China), F4/80 (Cell Signal Technology, MA, USA), CD4 (Cell Signal Technology, MA, USA), NF-*κ*B p65 (Cell Signal Technology, MA, USA), cleaved poly-ADP ribose polymerase (c-PARP) (Cell Signal Technology, MA, USA), glyceraldehyde 3-phosphate dehydrogenase (GAPDH) (Proteintech, Wuhan, China), phosphor-NF-*κ*B p65 (Cell Signal Technology, MA, USA), p-I*κ*B-*α* (Cell Signal Technology, MA, USA), and I*κ*B-*α* (Cell Signal Technology, MA, USA). TNF-*α*, IL-6, IFN-*γ*, and IL-1*β* enzyme-linked immunosorbent assay (ELISA) kits were purchased from DAKEWE Bioengineering (Shenzhen, China). Terminal deoxynucleotidyl transferase-mediated dUTP nick-end labeling (TUNEL) apoptosis assay kit and BeyoECL Plus were obtained from Beyotime Biotechnology (Shanghai, China). All other chemicals used were of highest analytical grade.

### 2.2. Animals

Male C57BL/6 mice (7-9 weeks old, 23–26 g) were obtained from SPF Biotechnology Co., Ltd. (Beijing, China). All animals were kept in a clean room at 24°C under a 12 h : 12 h light : dark cycle with free access to water and food. All animal experiments were approved by the Animal Care and Use Committee of Tongji Medical College of Huazhong University of Science and Technology.

### 2.3. Drug Administration

Con A was administered via the tail vein injection as described previously [16]. OI was dissolved in saline at a concentration of 1 mg/mL and was administered via intraperitoneal injection 2 h before Con A administration. ML385 was given once a day for 2 days via intraperitoneal injection. Mice were randomly divided into 6 groups (*n* = 6): (1) control group: mice were given vehicle (phosphate-buffered saline, PBS) during whole process; (2) OI group: mice received OI (100 mg/kg); (3) Con A group: mice received Con A (20 mg/kg); (4) low-dose OI group: OI (50 mg/kg)+Con A (20 mg/kg) group; (5) high-dose OI group: OI (100 mg/kg)+Con A (20 mg/kg); (6) ConA+OI+ML385 group: OI (50 mg/kg)+Con A (20 mg/kg)+ML385 (30 mg/kg). OI concentration was used according to previous study [17]. Animal samples of the liver and blood were collected 12 h after Con A treatment, according to our previous study [16]. We anaesthetized the mice with sodium pentobarbital to minimize the pain of the mice and collected approximately 500 *μ*L of blood through the eyeball. Then, the blood was subjected to high-speed centrifugation to isolate the serum for following experiments.

### 2.4. Cell Culture

The murine macrophage cell line RAW264.7 was purchased from the Cell Bank of the Chinese Academy of Science (Shanghai, China). The AML12 was purchased from Zhong Qiao Xin Zhou Biotechnology Co., Ltd (Shanghai, China). Both cell lines were cultured in DMEM supplemented with 10% fetal bovine serum. Logarithmic-phase AML12 and RAW264.7 cells were grown in 6-well plates as described previously [24]. The cells were pretreated with OI (100 *μ*M) for 2 h and then were treated with Con A (50 *μ*g/mL). Thereafter, the cells were harvested and used for subsequent experimental after Con A administration. Subsequently, we test the effect of OI in the presence of TNF-*α* on AML12 cells. AML12 cells were treated with TNF-*α* (100 ng/ml) and/or OI (100 *μ*M) for 24 h. Then, the apoptosis was measured by flow cytometry. For coculture experiments, we treated RAW264.7 cells with Con A and/or OI for 12 h and collected the supernatant, added it into AML12 cells for 24 h. The supernatants were collected to detect cytokine levels. Flow cytometry was used to detect AML12 cell apoptosis.

### 2.5. ALT and AST Assay

Serum was collected via centrifugation of blood sample at 1500 × g for 10 min. Subsequently, the activities of hepatic enzymes were measured according to manufacturer's instructions.

### 2.6. Histopathological Analysis and Immunohistochemical Analyses

Liver tissues were acquired and fixed in 4% paraformaldehyde for overnight. Subsequently, the tissues were embedded in paraffin, and 4 *μ*m thick sections were cut and stained with hematoxylin and eosin (H&E). Liver injury was evaluated based on the necrotic area according to the standard morphological criteria. Five random fields (200x) were selected, and the necrotic area was measured by using the ImageJ software. For immunohistochemical analysis, tissue sections were dewaxed in dimethyl benzene and rehydrated using graded alcohols. After recovering the antigen and blocking endogenous peroxidase, the sections were blocked in 5% BSA at 37°C for 30 min and incubated with primary antibody against cleaved poly-ADP ribose polymerase (1 : 200) overnight. Subsequently, horseradish peroxidase- (HRP-) conjugated secondary antibody and 3,3-diaminobenzidine tetrahydrochloride were added, and positive cells were visualized.

### 2.7. Immunofluorescence Staining

For hepatic tissues, the sections were dewaxed and processed as we described previously [16]. Subsequently, the primary antibody against F4/80 (1 : 200) was added and incubated overnight at 4°C. Then, sections were washed and incubated with a secondary antibody (ANT030s Alexa Fluor®594 Donkey anti-Rabbit IgG, 1 : 200). DAPI was added and incubated for 10 min to visualize the nucleus. For RAW 267.4 cells, the cells were washed and fixed in 4% paraformaldehyde for 15 min. The cells were treated with 0.1% TrixonX-100 for 10 min and blocked in 5% BSA for 2 h and incubated with antibodies against p65 (1 : 500) overnight at 4°C. Then, cells were washed and incubated with second antibody (ANT030s Alexa Fluor®594 Donkey anti-Rabbit IgG, 1 : 200). DAPI was incubated for 5 min to display nucleus. Five random fields (200x) were selected, and positive cells were measured by the ImageJ software.

### 2.8. TUNEL Assay

The tissue sections were dewaxed and rehydrated using xylene and graded ethanol. Subsequently, proteinase K (20 *μ*L/mL) without DNase was added at room temperature, and the sections were washed using PBS. Thereafter, the TUNEL detection mixture was added and incubated without light for 60 min at 37°C. DAPI was added for 10 min to visualize the nucleus. Five random fields (200x) were selected, and positive cells were evaluated using the ImageJ software.

### 2.9. Measurement of the GSH/GSSG Ratio and MDA Content

The hepatic tissues were collected and homogenized using PBS (weight/volume = 1 : 10). The supernatant was collected as described previously [16]. Subsequently, the GSH/GSSG ratio and MDA levels were evaluated according to the manufacturer's instructions, respectively.

### 2.10. ELISA Assay

Serum was acquired from the whole blood via centrifugation at 1500 × g for 10 min. The supernatant from RAW264.7 cells was collected 12 h after Con A treatment (50 *μ*g/mL). Subsequently, the levels of IL-6, TNF-*α*, IFN-*γ*, and IL-1*β* were measured according to the manufacturer's instructions, respectively.

### 2.11. Apoptosis Detected by Flow Cytometry

APC Annexin V with 7-AAD (BioLegend, USA) Apoptosis Detection Kit was used to detect the apoptosis. AML12 cells were collected after treatment, 100 *μ*l binding buffer was added to each tube, followed by APC, and 7-AAD reagent, and incubated for 25 min.

### 2.12. RNA Interference

The AML12 cells were grown in 6-well plates and cultured until the confluence reached 40-50%. The culture medium was replaced with a medium without antibiotics. The sequence of siRNA for Nrf2 was 5′-GCTCGCATTGATCCGAGATAT-3′. Transfection was performed using Lipofectamine® 6000 (Beyotime, Shanghai, China) according to the recommended instructions. Then, the culture medium was replaced with a complete medium, and the cells were used for subsequent experiments.

### 2.13. Immunoblotting

The total protein was extracted using RIPA lysate (#R0278, Sigma) according to the manufacturer's instructions. The BCA protein assay kit (Beyotime, Shanghai, China) was used to measure the protein concentration according to the manufacturer's instructions. An equal amount of protein was loaded onto sodium dodecyl sulphate-polyacrylamide gel electrophoresis, and immunoblotting was performed as described previously [24]. Primary antibodies against HO-1 (1 : 1000), Nrf2 (1 : 1000), Bax (1 : 1000), Bcl-2 (1 : 1000), NF-*κ*B p65 (1 : 1000), c-PARP (1 : 1000), GAPDH (1 : 2000), phosphor-NF-*κ*B p65 (1 : 1000), p-I*κ*B-*α* (1 : 1000), and I*κ*B-*α* (1 : 1000) were used. HRP-conjugated secondary antibodies were used to detect fluorescence using BeyoECL Plus as described previously [24].

### 2.14. Statistical Analysis

All data were expressed as the mean ± standard deviation. *p* value was calculated by one-way analysis of variance and followed by Tukey's post hoc test. *p* < 0.05 was considered statistically significant. All statistical analyses were performed using GraphPad Prism 8.

## 3. Results

### 3.1. Effects of OI on Con A-Induced Liver Damage in Mice

**Figure 1:**
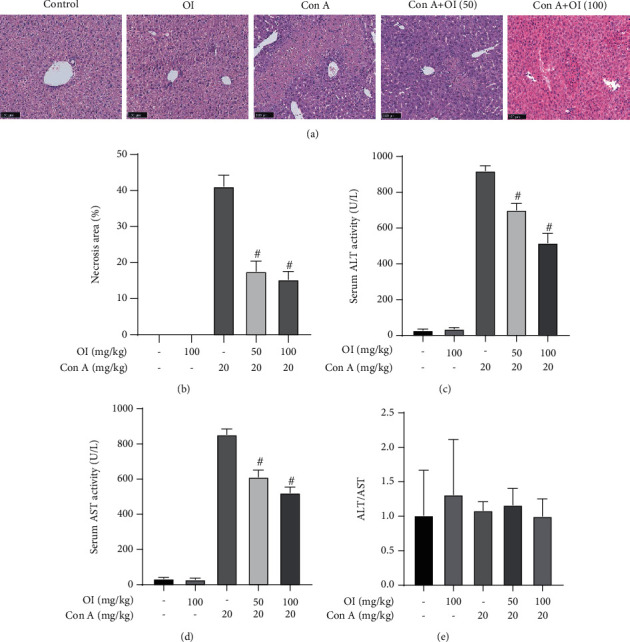
OI attenuated Con A-induced hepatic damage in mice. (a) Representative hepatic micrographs in control group, OI group (100 mg/kg), Con A group (20 mg/kg), low-dose OI group (OI: 50 mg/kg+Con A: 20 mg/kg), and Con A+OI group (OI: 100 mg/kg+Con A: 20 mg/kg). Then, we evaluated the (b) necrotic area, serum activities of (c) ALT and (d) AST, and (e) ALT/AST ratio of each group. Data are expressed as mean ± SEM (*n* = 6). ^#^*p* < 0.05 vs. Con A group.

As demonstrated in Figures 1(a) and 1(b), Con A injection caused significant histopathological damage. Five random fields were selected, and necrotic areas were measured by the ImageJ software. The necrotic area in the 50 mg/kg OI treatment group was lower than that of the Con A group. However, the dose of 100 mg/kg OI most significantly reduced the necrotic area. Furthermore, Con A significantly elevated the levels of ALT and AST, and both 50 mg/kg and 100 mg/kg OI treatment doses reduced the activity of ALT and AST (Figures 1(c) and 1(d)). Subsequently, we detected the ALT/AST ratio and found there was no significant difference among the groups (Figure 1(e)).

### 3.2. Negative Effects of OI Treatment on the Infiltration of Macrophages and Production of Cytokines

**Figure 2:**
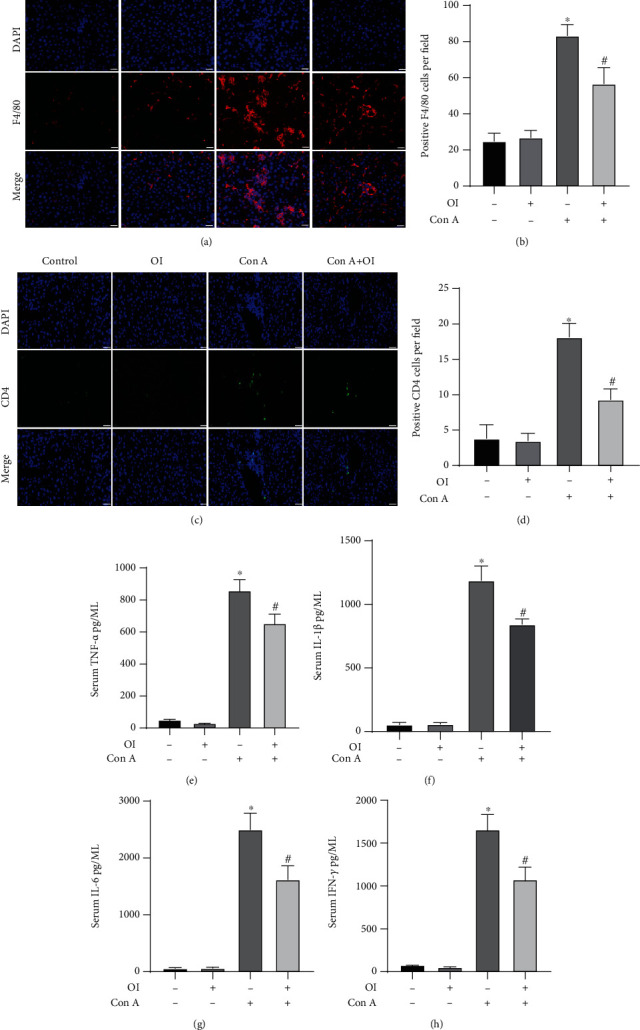
OI reduced Con A-induced macrophage infiltration and expression of proinflammatory cytokines in mice. (a) Representative immunofluorescence micrographs showed that the F4/80-positive cells in each group and the (b) positive cells in each group were evaluated. (c, d) Immunofluorescence for CD4-positive staining cells of the liver tissue. The serum levels of proinflammatory cytokines including (e) TNF-*α*, (f) IL-1*β*, (g) IL-6, and (h) IFN-*γ* were measured in each group. Data are expressed as mean ± SEM (*n* = 6). ^∗^*p* < 0.05 vs. control group. ^#^*p* < 0.05 vs. Con A group.

F4/80 is a widely used marker of liver macrophages [26]. We found that infiltration of F4/80-positive cells increased after Con A treatment. However, OI administration significantly reduced the number of F4/80-positive cells (Figures 2(a) and 2(b)). We labeled CD4^+^ T lymphocytes with CD4 and found that the number of CD4 fluorescence-positive cells in the Con A group significantly increased, while 4-OI attenuated this change (Figures 2(c) and 2(d)). Additionally, ELISA assay revealed that Con A administration markedly elevated the levels of TNF-*α*, IL-6, IFN-*γ*, and IL-1*β*, whereas OI administration significantly decreased the levels of TNF-*α*, IL-6, IFN-*γ*, and IL-1*β* (Figures 2(e)–2(h)). In addition, OI treatment alone altered neither the number of F4/80, CD4-positive cell number nor the expression of TNF-*α*, IL-6, IFN-*γ*, and IL-1*β*.

### 3.3. OI Attenuated Hepatocyte Death and Oxidative Stress in Con A-Treated Mice

**Figure 3:**
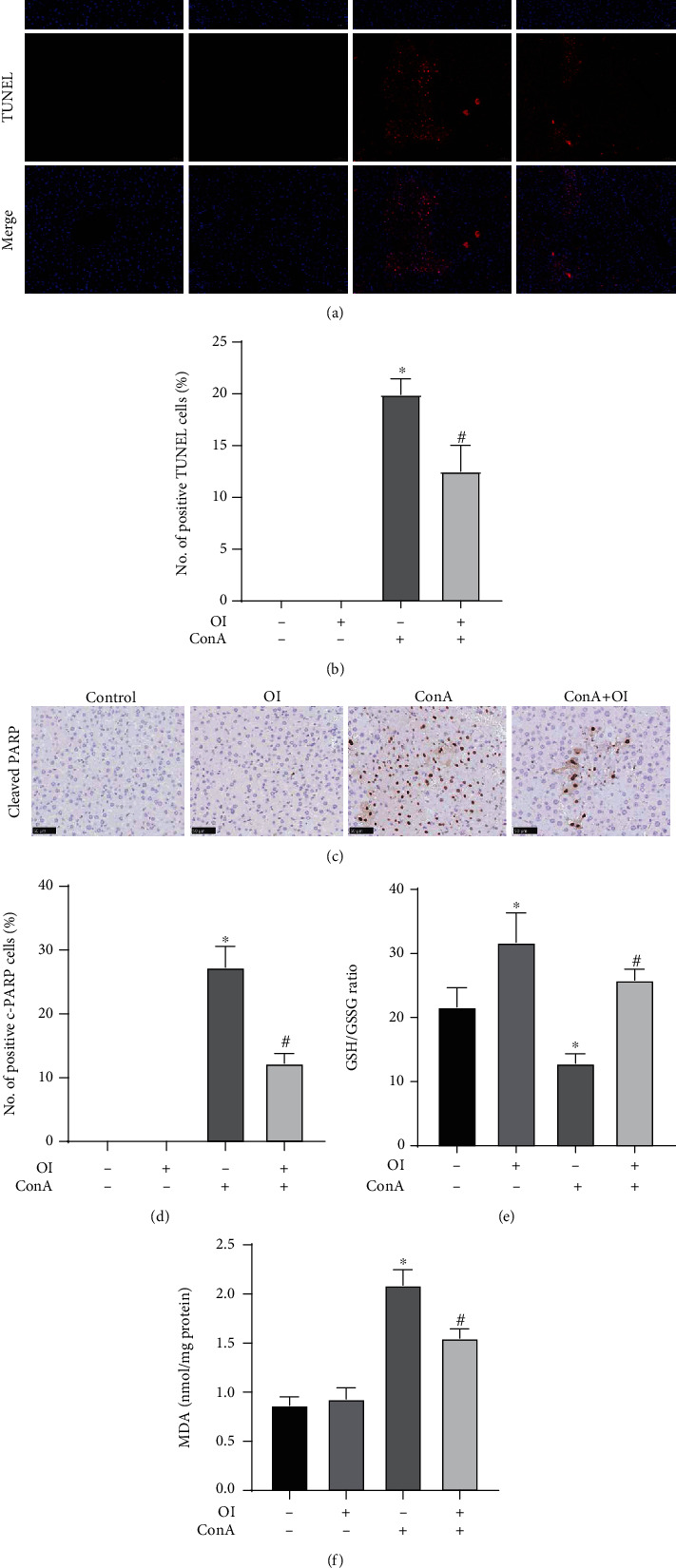
OI decreased Con A-induced hepatocyte apoptosis and oxidative stress in liver. Representative images for (a, b) positive TUNEL cells and immunohistochemical assay of (c, d) c-PARP were evaluated in hepatic tissues in each group. The (e) GSH/GSSG ratio and (f) MDA levels were measured in hepatic tissues in each group. Data are expressed as mean ± SEM (*n* = 6). ^∗^*p* < 0.05 vs. control group. ^#^*p* < 0.05 vs. Con A group.

Hepatocyte death is frequently used to evaluate the extent of liver injury. The TUNEL staining was used to detect the apoptosis in the Con A-induced hepatic injury model. Moreover, c-PARP is considered as a marker of apoptosis in Con A-induced liver injury [16]. We found that Con A administration significantly increased the number of TUNEL- and c-PARP-positive cells in liver tissues (Figures 3(a)–3(d)) compared with the control group. However, OI administration markedly reduced the number of TUNEL- and c-PARP-positive cells in Con A-treated mice. In addition, the GSH/GSSG ratio is the main dynamic index of the redox state of cells, and MDA content is a common index to evaluate membrane lipid peroxidation [27]. We measured the GSH/GSSG ratio and MDA content in liver tissues to assess the oxidative stress and found that the GSH/GSSG ratio was significantly reduced in Con A-treated mice. However, OI administration markedly increased the GSH/GSSG ratio in Con A-treated mice (Figure 3(e)). Moreover, the MDA content dramatically increased after Con A treatment and significantly decreased after subsequent OI treatment (Figure 3(f)).

### 3.4. OI Inhibited the Apoptosis of AML12 Cells by Activating Nrf2

**Figure 4:**
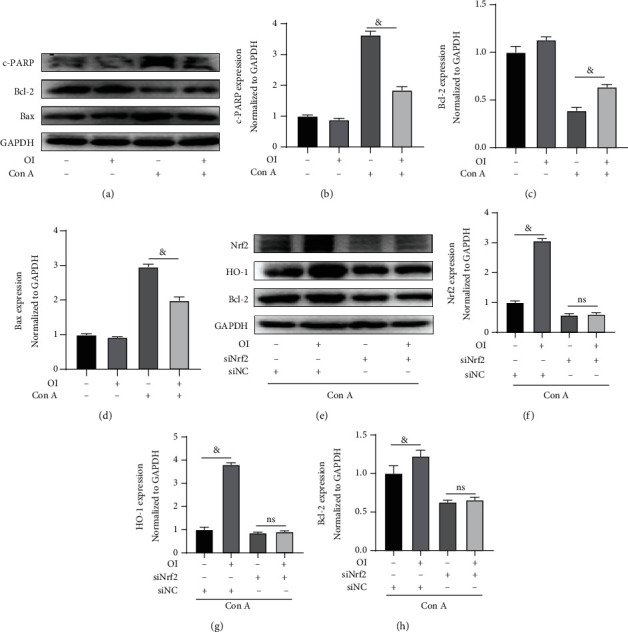
Nrf2 deletion reduced the protective effect of OI in Con A-treated AML12 cells. (a) The immunoblots of c-PARP, Bcl-2, and Bax in each group. The expression of (b) c-PARP, (c) Bcl-2, and (d) Bax was measured in each group. (e) The immunoblots of Nrf2, HO-1, and Bcl-2 in Con A with/without OI-treated AML12 cells with siNC or siNrf2 treatment. The expressions of (f) Nrf2, (g) HO-1, and (h) Bcl-2 were evaluated in each group. GAPDH was used for endogenous control. All tests were independently performed three times. ^&^*p* < 0.05. Data are expressed as mean ± SEM.

PARP cleavage promotes cell disintegration and can be used as a marker of apoptosis [28]. Bax is a key component of mitochondrial stress-induced apoptosis [29]. Bcl-2 exerts its antiapoptotic function by inhibiting the release of mitochondrial cytochrome [30]. In this study, Con A increased the expression of c-PARP and Bax and inhibited the expression of Bcl-2 in AML12 cells. However, OI treatment significantly reduced the expression of c-PARP and Bax and simultaneously increased the expression of antiapoptotic Bcl-2 (Figures 4(a)–4(d)). To verify if Nrf2 is associated the protective effects, we used the specific siRNA to knock down Nrf2 expression in AML12 cells, and siNC was used as a control. Under Con A treatment, OI significantly increased the expression of Nrf2 and its target gene HO-1 in the presence of Nrf2. However, after Nrf2 expression was inhibited, the expression of Bcl-2 was not increased with OI administration (Figures 4(e)–4(h)).

### 3.5. Protective Effects of OI Were Inhibited by ML385

**Figure 5:**
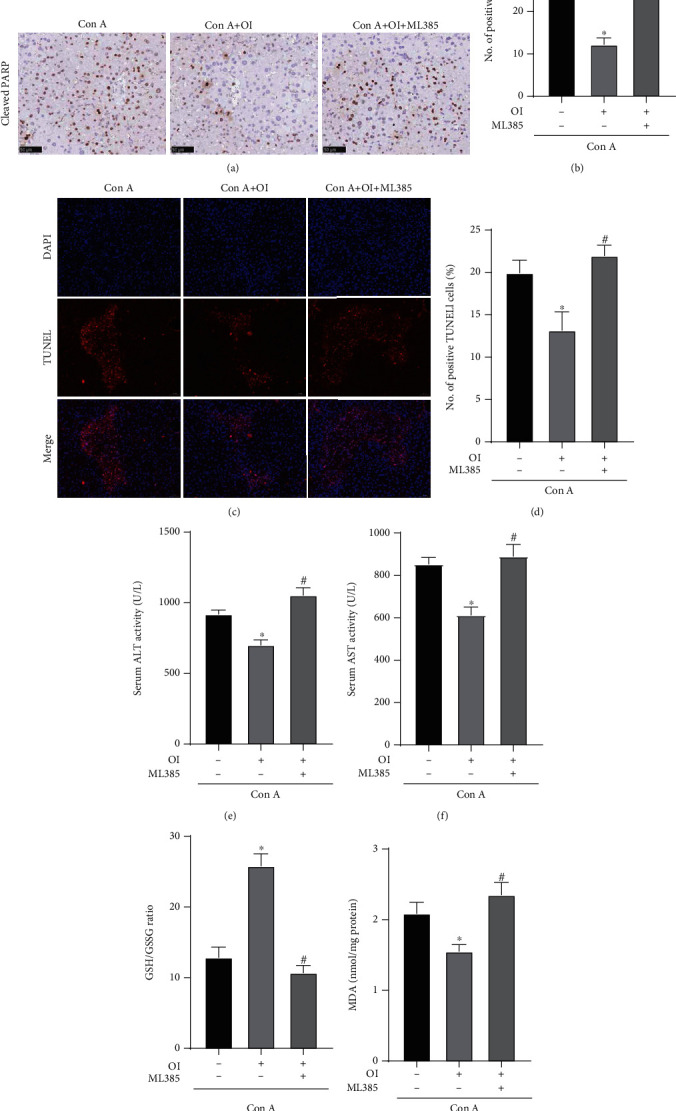
Protective effect of OI was decreased by ML385 in Con A-induced liver injury. (a, b) Immunohistochemical assay of c-PARP was evaluated in hepatic tissues in each group. (c, d) The hepatocyte apoptosis was evaluated in hepatic tissues in each group by TUNEL assay. The (e) serum ALT and (f) serum AST activities were evaluated in each group in mice. To evaluate the oxidative stress, the (g) GSH/GSSG ratio and (h) MDA level were measured in each group. Data are expressed as mean ± SEM (*n* = 6). ^∗^*p* < 0.05 vs. Con A group. ^#^*p* < 0.05 vs. Con A+OI.

We used ML385, a specific Nrf2 inhibitor, to assess whether the protective effect of OI was associated with Nrf2 activation in the AIH model. As shown in Figures 5(a)–5(f), OI exerted protective effects in Con A-treated mice, including reducing the number of c-PARP- and TUNEL-positive cells, and the activity of ALT and AST. However, ML385 administration in Con A+OI-treated mice increased the number of c-PARP- and TUNEL-positive cells and the activity of ALT and AST. Moreover, OI administration markedly increased the GSH/GSSG ratio and reduced MDA levels in Con A-treated mice. However, the GSH/GSSG ratio was decreased, and MDA content was increased in the ML385+Con A+OI group compared with the Con A+OI group (Figures 5(g) and 5(h)).

### 3.6. OI Inhibited the Activation of NF-𝜅B and Expression of Proinflammatory Mediators and Promoted the Nrf2/HO-1 Signaling Pathway

**Figure 6:**
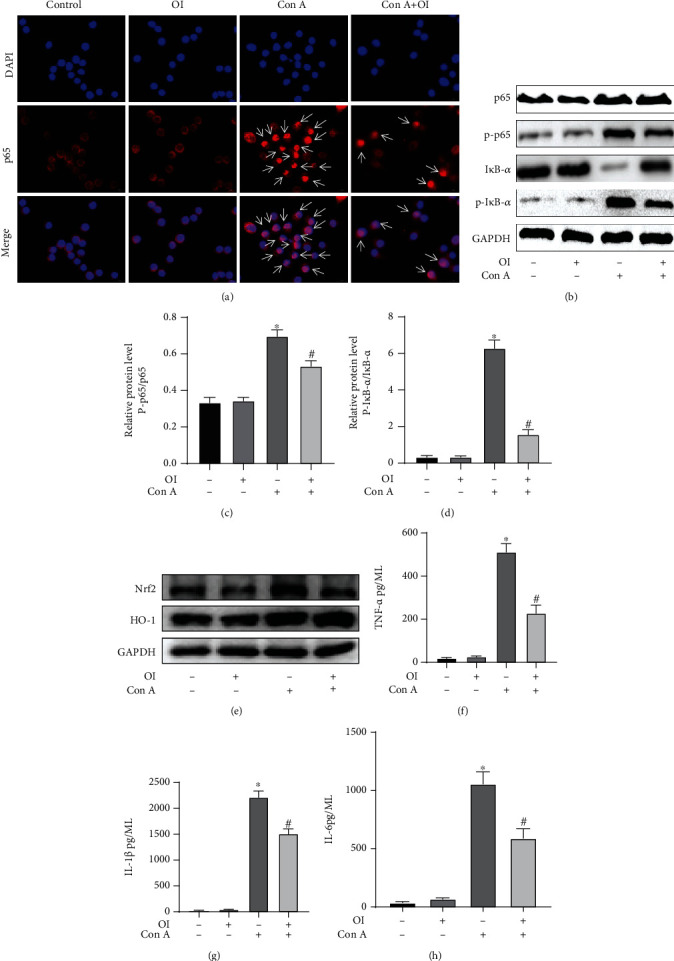
OI mitigated Con A-induced activation of NF-𝜅B activation and expression of proinflammatory cytokines in macrophages. (a) OI administration reduced Con A-induced NF-𝜅B p65 nuclear translocation by immunofluorescence in RAW264.7 cells. (b) Immunoblots of NF-𝜅B p65, phosphate-p65 (p-p65), I*κ*B-*α*, and phosphate-I*κ*B-*α* (p-I*κ*B-*α*) in RAW264.7 in each group. The relative expression of (c) p-p65/NF-𝜅B p65 ratio and (d) p-I*κ*B-*α*/I*κ*B-*α* ratio was analyzed in each group. (e) Western blot showed the expression levels of Nrf2 and HO-1. The levels of cytokines including (f) TNF-*α*, (g) IL-1*β*, and (h) IL-6 in supernatant were measured. GAPDH was used for endogenous control. White arrow showed positive nuclear translocation cells. All tests were independently performed three times. ^∗^*p* < 0.05 vs. control group; ^#^*p* < 0.05 vs. Con A group. Data are expressed as mean ± SEM.

Macrophage activation contributes to liver damage in Con A-induced AIH models and is associated with NF-𝜅B activation and the expression of proinflammatory such as TNF-*α*, IL-6, and IFN-*γ*. Our results revealed that Con A promoted the nuclear translocation and phosphorylation of NF-𝜅B p65 (Figures 6(a) and 6(b)) and phosphorylation of I*κ*B-*α* (Figure 6(b)) and increased the production of TNF-*α*, IL-6, and IFN-*γ* (Figures 6(f)–6(h)) in RAW264.7 cells. And we found that OI inhibited the Con A-induced nuclear translocation and phosphorylation of NF-𝜅B p65 (Figures 6(a) and 6(b)) and phosphorylation of I*κ*B-*α* (Figure 6(b)) and inhibited the expression of TNF-*α*, IL-6, and IFN-*γ* (Figures 6(f)–6(h)). Meanwhile, OI promoted the expression of Nrf2 and HO-1, thus inhibiting the inflammatory response (Figure 6(e)).

### 3.7. OI Inhibited TNF-*α*, and Supernatants from Con A-Treated RAW264.7 Cells Induced Hepatocyte Apoptosis

**Figure 7:**
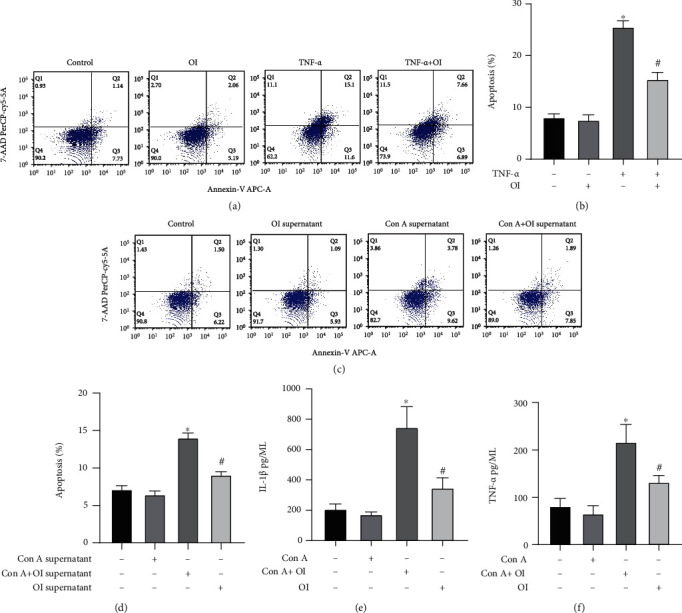
OI inhibited TNF-*α*, or supernatants from Con A-treated RAW264.7 cells induced hepatocyte apoptosis. (a, b) TNF-*α* significantly induced apoptosis in hepatocytes, whereas OI attenuated this change. RAW264.7 cells were treated with Con A and/or OI for 12 h, and the supernatants were added into AML12 cells for 24 h. Flow cytometry was used to detect (c, d) hepatocyte apoptosis, and the supernatants were collected to detect the levels of (e) IL-1*β* and (f) TNF-*α*. ^∗^*p* < 0.05 vs. control group; ^#^*p* < 0.05 vs. Con A or TNF-*α* group. Data are expressed as mean ± SEM.

We analyzed the effect of OI in the presence of TNF-*α* and found that TNF-*α* significantly induced apoptosis in hepatocytes, whereas OI attenuated these changes (Figures 7(a) and 7(b)). To further assess the effect of macrophages and OI on Con A-induced AIH, we treated RAW264.7 cells with Con A and/or OI for 12 h and collected the supernatant, added into AML12 cells for 24 h, and collected the supernatant to detect cytokines levels. Flow cytometry was used to detect apoptosis. The results revealed that apoptosis was more evident in the Con A group than in the control group and significantly decreased in the Con A+OI group compared with the Con A group (Figures 7(c) and 7(d)). Simultaneously, we detected the level of cytokines (IL-1*β* and TNF-*α*) in the supernatant and found OI inhibited the release of IL-1*β* and TNF-*α* induced by Con A (Figures 7(e) and 7(f)).

## 4. Discussion

The liver is a pivotal organ of the digestive system. Multiple toxic compounds can cause liver injury. Con A is a lectin extracted from jack-bean and enhances T cell proliferation in response to mitogens. By administering Con A through tail vein in mice, Tiegs et al. [31] established a reliable experimental animal model to study the pathological process of AIH. In recently years, studies have demonstrated that itaconate, an intermediate metabolite of the tricarboxylic acid cycle, exerted protective effects on several disease models by regulating inflammation and redox balance [18]. OI, a cell-permeable mimic of itaconate, is considered as a suitable mimic of itaconate and has strong anti-inflammatory and protective effects on the pathology of several diseases [32–34]. For example, OI mitigated hepatic damage in liver ischemia-reperfusion injury by activating the Nrf2 pathway [25]. In addition, we previously found that OI inhibited carbon tetrachloride-induced liver injury by reducing inflammation and oxidative stress [24]. However, the effect of OI on AIH model has not been investigated. In the present study, we found OI exhibited protective effects on Con A-induced hepatic damage in mouse AIH model. Our results revealed that OI treatment ameliorated Con A-induced liver injury by reducing inflammatory response, oxidative stress, and apoptosis, and the underlying mechanism was associated with Nrf2 signaling activation and NF-*κ*B pathway inhibition.

Serum ALT and AST levels are usually used to assess the liver damage. These transaminases are released into blood during hepatocyte damage. In the present study, Con A-induced hepatic injury was successfully elicited, which was evidenced by histopathological examination and elevation of ALT and AST levels. OI treatment reduced the necrotic area in liver tissues and serum ALT and AST levels in the AIH mouse model. ALT/AST ratio, a parameter widely used in the clinic, can be applied to autoimmune liver diseases including autoimmune hepatitis. However, our study showed that AST/ALT ratio was not affected with OI administration. Maybe the ALT/AST ratio did not apply to Con A-induced mouse AIH model. Furthermore, inflammatory cells play an important role in AIH-associated hepatic injury. Regulatory T cells (Tregs) are one of the important factors that maintain immune tolerance. Loss of Tregs in mice has been reported as a method to induce AIH, leading to hepatic injury [35]. Moreover, activation of the KCs, also called hepatic macrophages, is involved in Con A-induced hepatic injury in mice [36]. In our previous study, we also found that Con A-induced liver damage was associated with the accumulation of macrophages and CD4^+^ T cells [16]. Therefore, alteration of immune cells is pivotal in the development of AIH. Here, we used F4/80 to mark macrophages in liver tissues and found that OI pretreatment significantly reduced macrophage infiltration caused by Con A. In addition, excessive cytokines are involved in the pathogenesis of Con A-induced liver injury. Proinflammatory cytokines such as TNF-*α* and INF-*γ* are crucial in Con A-induced hepatic injury [37]. Macrophages produce TNF-*α*, which is an important signal for apoptosis [38]. INF-*γ*, a pivotal mediator in the progression of AIH [39], can synergize with TNF-*α* to increase the production of other inflammatory mediators, thus promoting Con A-induced liver injury. In the present study, OI inhibited the levels of TNF-*α*, IL-1*β*, IL-6, and INF-*γ* in Con A-treated mice. Therefore, these findings indicated that OI ameliorated Con A-induced liver damage by inhibiting inflammatory responses.

In addition to inflammation, oxidative stress can cause liver injury. Excessive ROS production is the main cause of oxidation and leads to destruction of proteins, DNA, and lipid [40]. Accumulation of ROS in the liver induces cell death and eventually causes hepatic damage. Several antioxidative systems, such as Nrf2, can activate antioxidative enzyme expression to maintain the cellular redox balance to avoid cell damage [41]. Nrf2 is anchored in the cytoplasm by Keap1, which promotes the ubiquitination of Nrf2 and leads to rapid degradation by proteasomes. When cells are attacked by ROS or electrophilic molecules, Nrf2 dissociates from Keap1 and translocates into the nucleus. Nrf2 initially forms a heterodimer with small Maf proteins and subsequently combines with ARE to activate the expression of antioxidant enzymes regulated by Nrf2 [42]. We have previously confirmed that Con A significantly increased ROS levels in hepatocytes and promoted liver damage [16]. c-PARP is validated as reliable marker for apoptosis [43]. In the present study, we found that OI administration reduced the number of positive TUNEL- and c-PARP-positive cells in liver tissues. Therefore, this finding suggested that OI attenuated Con A-induced hepatocyte death. Moreover, MDA is a product of lipid peroxidation, which can cause cytotoxicity via cross-linking polymerization of proteins, nucleic acids, and other life macromolecules [44]. GSH is an important antioxidant molecule that eradicates ROS [45]. Increasing the GSH/GSSG ratio is important for maintaining the integrity of the antioxidant and detoxifying its function [45]. Therefore, both MDA content and GSH/GSSG ratio are pivotal indices to evaluate the oxidative stress in the liver. In this study, we found that MDA level markedly reduced and the GSH/GSSG ratio was increased with OI treatment in Con A-treated mice. Therefore, protective effects of OI on AIH model were partially associated with its antioxidative property.

It has been reported that the protective effects of OI in liver diseases are mainly associated with Nrf2 activation [24]. Activation of Nrf2 can promote multiple detoxicating and antioxidant genes against oxidative stress and inflammation. For example, OI prevents lung damage caused by methicillin-resistant staphylococcus aureus bacteremia by activating the Nrf2/ARE pathway. This protective effect was abolished after ML385 administration or Nrf2 deletion in mice [46]. In our study, we further explored whether OI protected hepatocytes from apoptosis by activating Nrf2. We used Bcl-2 to evaluate the apoptosis of AML12 cells in vitro. Bcl-2 can form a dimer with the proapoptotic protein Bax. If the relative amount of Bax is higher than that of Bcl-2, the level of Bax homodimer increases, thus promoting cell death [47]. If the relative amount of Bcl-2 is higher than that of Bax, the formation of Bcl-2/Bax heterodimer will be promoted and the amount of Bcl-2 homodimer will be increased to inhibit the apoptosis [47]. First, we found that OI decreased the level of c-PARP and Bax in Con A-treated AML12 cells, and the Bcl-2 expression was increased by OI. After Nrf2 was knocked down, OI could neither increase the expression of HO-1 nor Bcl-2 in vitro. These findings revealed that the protective effect of OI on hepatocytes was associated with Nrf2 activation. Moreover, we blocked Nrf2 activation in vivo using ML385, a specified Nrf2 inhibitor, which reduced the protective effect of OI. We found that the indices of apoptosis, including the number of c-PARP- and TUNEL-positive cells, were increased after ML385 administration in vivo. In addition, the levels of hepatic enzymes and oxidative stress markers were increased after ML385 administration in Con A-treated mice. Therefore, at least in part, OI mitigated the Con A-induced hepatic damage by activating Nrf2, thus inhibiting inhibited excessive oxidative stress and apoptosis.

Hepatic macrophages play a crucial role in the pathogenesis of Con A-induced AIH by producing excessive inflammatory mediators such as TNF-*α*, which may cause hepatocyte damage [37]. NF-𝜅B signaling is an important transcription factors that regulates inflammatory responses, cell apoptosis, and stress responses [48]. Normally, NF-𝜅B p65 is combined with I*κ*B-*α* in cytoplasm. Inflammatory stimuli, such as LPS, can initiate phosphorylation and degradation of I*κ*B-*α*. Subsequently, NF-𝜅B p65 dissociates and translocates into the nucleus to enhance proinflammatory cytokine expression [49]. Meanwhile, NF-𝜅B p65 is phosphorated and promotes the transcription of inflammatory mediators. We have previously validated that Con A caused liver injury by overactivating NF-𝜅B p65 to induce excessive inflammation [16]. In the present study, OI inhibited Con A-induced nuclear translocation and phosphorylation of NF-𝜅B p65 and phosphorylation and degradation of I*κ*B-*α*. Furthermore, OI can activate Nrf2 and HO-1, which inhibit inflammatory response. Additionally, we found OI inhibited the expression of proinflammatory cytokines in Con A-treated macrophages. These indicated that the protective effect of OI was associated with inhibition of the NF-𝜅B pathway activation.

## 5. Conclusion

Our study reveals that OI exerts protective effects in Con A-induced AIH by mitigating inflammation, oxidative stress, and hepatocyte death. OI reduces oxidative stress and inhibits the hepatocytes apoptosis by activating the Nrf2 signaling pathway to alleviate hepatic damage. In addition, it significantly inhibits the activation of NF-𝜅B in macrophages to attenuate the inflammatory response, thus eventually alleviating liver damage. Therefore, our study assessed the roles of OI in Con A-induced AIH and provided evidence that OI might be a potential therapeutic drug for AIH. Of course, more studies might be needed to do and clarify the effect of OI is only partial.

## Data Availability

The data used to support the findings in this study are available from the corresponding author upon reasonable request.
